# A Comparative Study on a Novel Quality Assessment Protocol Based on Image Analysis Methods for Color Doppler Ultrasound Diagnostic Systems

**DOI:** 10.3390/s22249868

**Published:** 2022-12-15

**Authors:** Giorgia Fiori, Andrada Pica, Salvatore Andrea Sciuto, Franco Marinozzi, Fabiano Bini, Andrea Scorza

**Affiliations:** 1 Department of Industrial, Electronic and Mechanical Engineering, University of Roma Tre, 00146 Rome, Italy; 2 Department of Mechanical and Aerospace Engineering, “Sapienza” University of Rome, 00184 Rome, Italy

**Keywords:** quality assessment, color doppler, ultrasound diagnostic systems, flow phantom, test parameters, image analysis methods, Kiviat diagram

## Abstract

Color Doppler (CD) imaging is widely used in diagnostics since it allows real-time detection and display of blood flow superimposed on the B-mode image. Nevertheless, to date, a shared worldwide standard on Doppler equipment testing is still lacking. In this context, the study herein proposed would give a contribution focusing on the combination of five test parameters to be included in a novel Quality Assessment (QA) protocol for CD systems testing. A first approach involving the use of the Kiviat diagram was investigated, assuming the diagram area, normalized with respect to one of the gold standards, as an index of the overall Doppler system performance. The QA parameters were obtained from the post-processing of CD data through the implementation of custom-written image analysis methods and procedures, here applied to three brand-new high-technology-level ultrasound systems. Experimental data were collected through phased and convex array probes, in two configuration settings, by means of a Doppler flow phantom set at different flow rate regimes. The outcomes confirmed that the Kiviat diagram might be a promising tool applied to quality controls of Doppler equipment, although further investigations should be performed to assess the sensitivity and specificity of the proposed approach.

## 1. Introduction

Ultrasound (US) is an interactive non-invasive imaging technique that provides quantitative information on anatomical districts through the propagation of ultrasound waves in soft tissues. Major US advantages compared with other imaging techniques, e.g., computed tomography or magnetic resonance, are its ease of use, real-time imaging, cost-effectiveness, portability, and patient safety [1,2]. In the last decades, active research in the US field has led to advancements in transducer technology and digital electronics with a consequent improvement of diagnostic information content [2,3]. Therefore, the US technique is applied by clinicians from different medical fields to provide diagnosis and treatment [4,5,6,7,8,9]. As a consequence, the use of US devices increased in recent years, and the worldwide market for medical ultrasound is projected to reach USD 8.4 billion in 2023, with an average annual growth rate of roughly 5.9% [10].

Color Doppler (CD) imaging, developed in the 1980s, allows the 2D real-time representation of blood flow superimposed on the anatomical image [1,2,11,12,13]. A color map codes and quantifies the velocity of blood flow inside a region of interest (or color box) adjusted by the operator on the B-mode grayscale ultrasound image as a function of the clinical requirements. Currently, CD is among the most widely used techniques in the medical field [1,2] since it is a powerful tool that allows hemodynamic monitoring and the visualization of the flow patterns in blood vessels. However, in the scientific community, controversy about whether the CD technique provides qualitative diagnostic data—non-repeatable and subjective estimations—rather than quantitative information—repeatable and objective measurements—of flow velocity still exists. This disagreement may be justified by high CD measurement uncertainties that can reach up to 50% [14]. Moreover, it is worth pointing out that a commonly accepted worldwide standard for Doppler ultrasound equipment testing has not been developed yet [15,16,17]. Attempts to define theoretical and experimental methods for medical US equipment Quality Assessment (QA) were made by several national and international organizations [16] over the years, with the consequent investigation of suitable tests for B-mode imaging, as well-documented in the literature [18,19,20,21,22,23]. Among these professional organizations, the American Institute of Ultrasound in Medicine (AIUM), the American Association of Physicists in Medicine (AAPM), the American College of Radiology (ACR), the European Federation of Societies for Ultrasound in Medicine and Biology (EFSUMB), and the Institute of Physics and Engineering (IPEM) are included.

Nowadays, although the demand for proper QA protocols has increased in the last years [16,24,25,26,27,28], performance evaluation of Doppler systems is still an open issue in the scientific research field. This is mostly due to the lack of consensus among the professional bodies about the US system configuration settings, as well as which and how many quality parameters to be processed and included in a Quality Control (QC) program for Doppler testing [16]. In this regard, the wide range of Doppler performance parameters proposed in the literature [16,25] often represents a considerable burden that requires an approach summarizing all their contributions in a few meaningful quantities that can be easily and quickly interpreted by the technician. This critical aspect is very common in several scientific fields where an effective representation of multivariate data is needed, and it is often achieved by means of a Kiviat diagram (or Kiviat plot, spider plot) [29,30]. This type of plot is characterized by a series of spokes projecting from a center point, with each spoke representing a different variable axis. The values of the variables are encompassed into the spoke length, and the plotted values are connected to form a polygon. The shape of the Kiviat diagram makes it easy to visualize and useful to compare different variables in a single graphical plot, especially when a reference or gold standard polygon is included. Nowadays, it is considered a useful comparative tool for outcome metrics since it allows both to convey a large amount of information and provide a standardized overview of different indicators [29,31]. In this regard, the Kiviat diagram could be a promising tool also in the assessment of Doppler system performance by integrating the outcomes of multiple meaningful test parameters.

Kiviat diagrams were introduced in the 1980s as a means for monitoring computer system hardware performance, and, to date, they are commonly used in several fields such as social sciences, economics, engineering, computing, and information technology and are mostly used as a tool for comparing performance metrics [29]. Although the use of the Kiviat plot in health-related literature is not so widespread, some examples should be mentioned. For instance, Kiviat plots have found utility in presenting data related to performance benchmarking at the patient and hospital levels for orthopedics surgery [31] or diagnostic performance of ultrasonography in patients with pneumonia [32].

From the above considerations, the aim of the present study is to propose and investigate the first approach to the effective combination of five parameters to be included in a novel QA protocol for Color Doppler diagnostic systems based on Kiviat diagrams. The proposed approach would give a contribution to the field since it allows quantifying the overall Doppler performance of US systems according to a probe-setting pair. Performance data could be used both to compare US systems manufactured by different companies and monitor Doppler system degradation over time. The latter usually occurs as a slow and progressive worsening of the image quality that could negatively affect the accuracy and efficacy of clinical diagnosis [33,34].

Three brand-new ultrasound systems, each of them equipped with a phased and convex array probe, were tested in two configuration settings. In this first comparative study, CD performance was evaluated in terms of: blind angle [35], registration error [36], average maximum velocity sensitivity [37], velocity measurements accuracy, and temporal resolution. These performance parameters, derived from QC tests already proposed in the literature [16] and recommended by international organizations [38,39], allow for quantifying Color Doppler functionality. They were obtained from the post-processing of Color Doppler data by means of automatic and objective image analysis procedures, whose measurement uncertainty contribution was estimated through the implementation of Monte Carlo Simulations (MCSs). One of the main advantages of the methods proposed is the possibility to overcome the intrinsic limits of visually-assessed performance tests since several test parameters recommended by the abovementioned professional organizations are qualitatively defined and suffer from operator-related errors [10,28,38,39].

The study herein proposed is organized as follows: Section 2 deals with the experimental setup adopted, the QA test parameters definition and description, as well as the normalization procedure proposed to combine and compare the outcomes retrieved. In Section 3, the measurement uncertainty analysis of the implemented image analysis-based methods through MCSs is carried out. In Section 4, experimental results are presented. In Section 5, the obtained outcomes are discussed, and future research directions are highlighted. Finally, the conclusions are outlined in Section 6.

## 2. Materials and Methods

### 2.1. Experimental Setup

The experimental setup included three high technology level US diagnostic systems, equipped with a phased (P) and a convex (C) array probe each, and a commercial Doppler flow phantom [40], whose specifications are reported in Table 1. The reference test device is constituted by a horizontal and diagonal vessel of known and constant cross-sectional area filled with blood-mimicking fluid. The flow rate can be adjusted to provide constant or pulsatile flows in the range of 1.7–12.5 mL·s^−1^.

The two US probe models mounted on each diagnostic system were set at the best configuration settings as suggested by the product specialists (configuration A) and by reducing both pre- and post-processing settings (configuration B) to allow the comparison of the results retrieved from different US systems at similar working conditions [36,37,41,42,43]. The main settings for both configurations are listed in Table 2. The three high technology level US systems, produced by different manufacturers, were anonymously addressed as system 1, system 2, and system 3. Experimental data were acquired on a portion of the diagonal vessel for the estimation of all test parameters (higher field of view setting, FOV_1_), except for the blind angle. In this case, acquisitions were carried out on a portion of the horizontal vessel (lower field of view setting, FOV_2_).

Color Doppler videos lasting 3 s were collected at the lowest Doppler frequency of each probe. Since the frame rate varies according to the US system and probe used, as well as to several pre- and post-processing settings (e.g., field of view, color box size, and position), a different total number of frames was acquired for each video. Therefore, a fixed number of frames *n_fr_* was selected for the two probe models as the minimum number of frames acquired in 3 s, that in this study corresponds to *n_fr_* = 30 and *n_fr_* = 24 for the phased and the convex array probe, respectively. This choice allowed the comparison of the outcomes retrieved from different US systems equipped with the same probe model. In this regard, CD gain was properly set according to both the phantom flow rate and the color map scale for the assessment of all the test parameters except for the registration error. In the latter case, Color Doppler videos were acquired, increasing the CD gain control until the step before the electronic noise appeared on the US display.

Each QC parameter investigated in this study and described in the following subsection was tested at three distinct flow rate regimes (low *Q_L_*, medium *Q_M_*, and high *Q_H_*) set on the Doppler phantom (Table 3). In particular, for the average maximum velocity sensitivity parameter, the flow rate was adjusted at two constant values to achieve a flow step of 1.5 mL·s^−1^ for each regime. On the other hand, the temporal resolution parameter was tested on a single flow rate and by varying the Color Doppler line density setting (low *LD_L_*, medium *LD_M_*, and high *LD_H_*).

### 2.2. Test Parameters for QA Protocol

#### 2.2.1. Blind Angle

**Figure 1:**
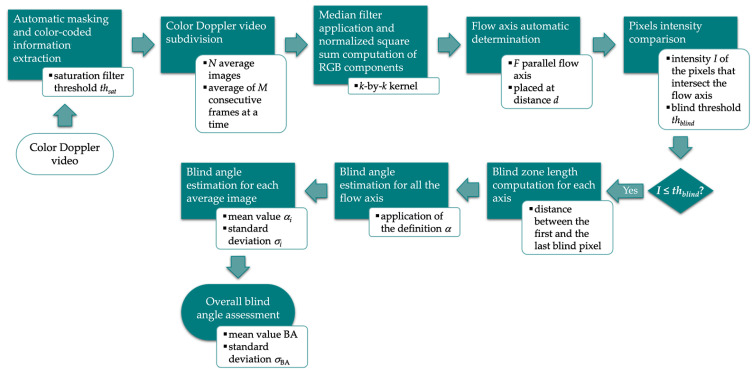
Flow chart of the image analysis-based method for blind angle parameter estimation.

The blind angle was defined in [35] as the range of beam angles for which the US probe is not able to detect flow velocities when the insonation angle approaches 90°. In this case, the Doppler signal is almost zero, and a black flow area is displayed in the color box. From this consideration, the blind angle *α* was mathematically expressed as follows:

(1) $$\alpha =2\arctan\left(\frac{a}{2h}\right)$$

where *a* is the blind zone transversal size, i.e., flow region in which no moving reflectors are detected, and *h* is the depth from the scanning surface. Such parameter was derived by considering directional accuracy at 90° performance test recommended by the AIUM [38] for Color Doppler QA. The international organization provides a qualitative definition of the test, which is performed through a visual inspection of the US system display. Conversely, the image analysis-based method, already proposed in [35], post-processes Color Doppler videos for the automatic and objective estimation of the blind angle parameter. It requires data to be collected on a portion of the Doppler phantom vessel displayed perpendicularly to the US propagation. The main steps of the measurement method, implemented by MATLAB software, are described in the following and shown in Figure 1.

**Figure 2:**
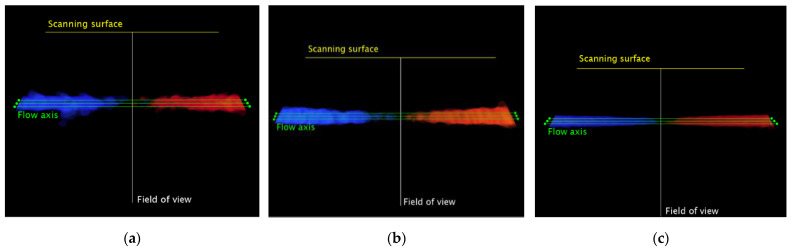
Flow axis placed on the *i*-th average image for (**a**) system one, (**b**) system two and (**c**) system three equipped with convex array probes in configuration B at medium flow rate regime *Q_M_*. Blue color indicates flow away from transducer, while red color indicates flow toward the transducer.

Automatic masking is applied to exclude the patient information and US settings details (US image extraction), while the color-coded information is extracted through a threshold-based saturation filter *th_sat_* [44]. Then, *N* average images are obtained by averaging *M* consecutive frames of the CD video, and a median filter with *k*-by-*k* kernel is applied to reduce color noise. After the computation of the normalized square sum of the RGB components, *F* parallel flow axis, placed at a fixed distance *d* among them, are automatically determined (Figure 2). At this point, the intensity *I* of each pixel intersecting the axis in the flow area at around 90° is compared with a blind threshold *th_blind_* for the computation of the blind zone length. The blind angle is estimated for all the flow axis as defined in Equation (1), and the mean value *α_i_* is computed together with its standard deviation *σ_α,i_*, obtaining a first estimation of the test parameter for each average image (*i* = 1, …, *N*). Finally, the overall blind angle BA and the corresponding standard deviation *σ*_BA_ are assessed as follows:

(2) $$\mathrm{BA}=\frac{1}{N}\sum_{i=1}^{N}\alpha_{i}$$

(3) $$\sigma_{\mathrm{BA}}=\frac{1}{N}\sqrt{\sum_{i=1}^{N}\sigma_{\alpha ,i}^{2}}$$

The method specifications assumed in this study are listed in Table 4.

#### 2.2.2. Registration Error

**Figure 3:**
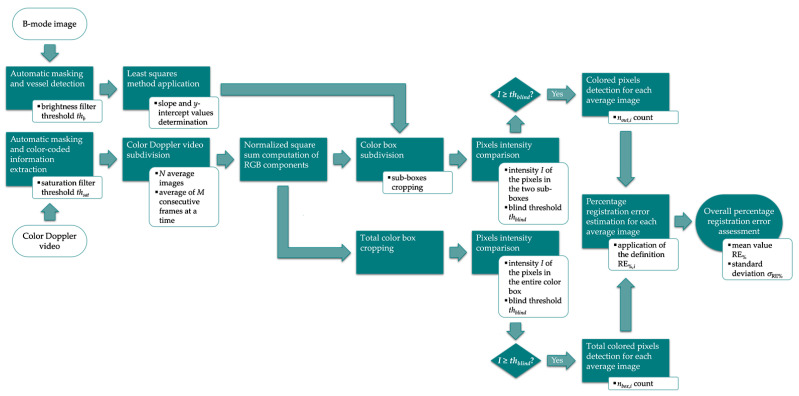
Flow chart of the image analysis-based method for registration error parameter estimation.

The Registration Error (RE) was defined in [36] as the degree of color bleeding when color write priority system control [1] is set to the maximum. It allows quantifying and monitoring the color flow misregistration, i.e., the positioning error of color flow information. The parameter was derived starting from Color/Power duplex priority control function included by the IPEM [39] among the basic functional checks for CD designed for control functioning assessment or faults detection. In this study, the semi-automatic method proposed in [36] was improved by removing the external action of the operator required to draw the vessel boundaries. The main steps of the improved method, implemented in MATLAB environment, are described in the following and shown in Figure 3.

The fully automatic method requires the processing of both a Color Doppler video and a B-mode image acquired on a Doppler flow phantom by maintaining the probe still on the scanning surface through a holder. Moreover, data need to be collected on a straight portion of the phantom vessel.

The US image extraction is applied to the grayscale anatomical image, and an adaptive threshold *th_b_* proportional to the mean brightness *V* (Value in the HSV hexcone model) [45,46] of the US image is automatically determined. This threshold works as a filter for the objective detection of the lumen as well as the boundaries of the phantom vessel. The least squares method is used to determine the slope and *y*-intercept values of the two straight lines that best approximate the upper and lower boundaries.

**Figure 4:**
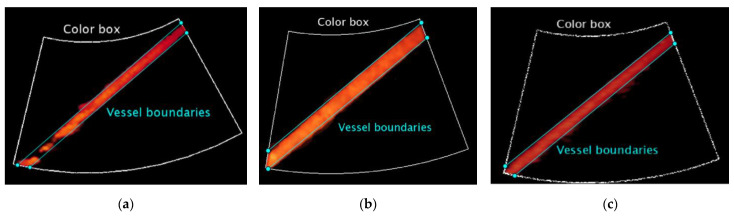
Straight lines approximating the upper and lower vessel boundaries used to subdivide the color box of the *i*-th average image for (**a**) system one, (**b**) system two and (**c**) system three equipped with convex array probes in configuration B at medium flow rate regime *Q_M_*.

On the other hand, the Color Doppler video is processed both through the US image extraction and a threshold-based saturation filter *th_sat_* [44] to extract the color-coded information. Then, *N* average images are obtained by averaging *M* consecutive frames, and the normalized square sum of the RGB components is computed. As in [36], the two abovementioned straight lines are used to subdivide and crop the color box of each average image into two different sub-boxes (Figure 4). At this point, the intensity *I* of each pixel in the sub-boxes is compared with the threshold *th_blind_* for the computation of *n_out_*, i.e., the number of colored pixels outside the vessel walls. The percentage registration error for each average image RE_%*,i*_ (*i* = 1, …, *N*) is estimated by applying the following mathematical expression [36]:

(4) $${\mathrm{RE}}_{\%,i}=\frac{n_{out,i}}{n_{box,i}}\cdot 100$$

where *n_box,i_* is the number of pixels in the entire color box of the *i*-th image whose intensity is above the threshold.

Finally, the overall percentage registration error RE_%_ is computed as the mean value of the *N* percentage registration values retrieved, and the standard deviation *σ*_RE%_ is estimated. According to Equation (4), the test parameter is expected to be 0%, i.e., no color flow misregistration.

The method specifications assumed in this study are listed in Table 5.

#### 2.2.3. Average Maximum Velocity Sensitivity

**Figure 5:**
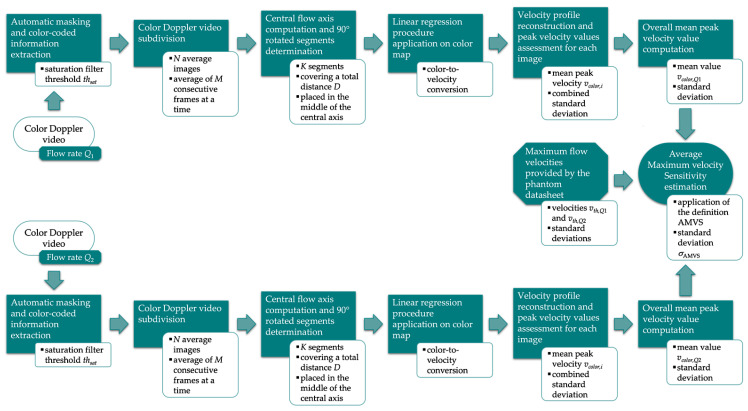
Flow chart of the image analysis-based method for AMVS parameter estimation.

The Average Maximum Velocity Sensitivity (AMVS) is a sensitivity test parameter defined and preliminarily investigated in [41] for Pulsed Wave Doppler QC and in [37] for Color Doppler QC. It allows quantifying the US system response to flow variations provided by a reference device. In this study, the method already proposed in [37] was improved in order to process the Color Doppler videos. The main steps of the updated method, implemented through a custom-written algorithm in MATLAB, are described in the following and shown in Figure 5.

**Figure 6:**
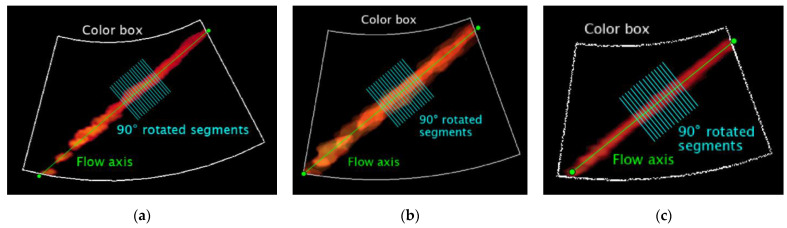
Ninety-degree rotated segments automatically drawn on the *i*-th average image for (**a**) system one, (**b**) system two and (**c**) system three equipped with convex array probes in configuration B at 7.0 mL·s^−1^.

**Figure 7:**
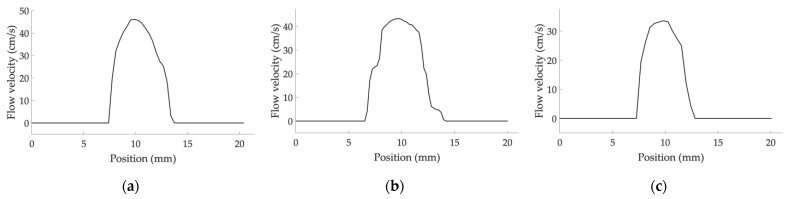
Example of reconstructed velocity profile associated with a single segment drawn on the *i*-th average image for (**a**) system one, (**b**) system two and (**c**) system three equipped with convex array probes in configuration B at 7.0 mL·s^−1^.

As already described for the previous parameters, the US image extraction is carried out, while the color-coded information is extracted through the threshold-based saturation filter *th_sat_* [44]. Then, *N* average images are obtained by averaging *M* consecutive frames of the Color Doppler video. At this point, the central flow axis is determined to automatically draw *K* segments rotated at 90° covering a total distance *D* and placed in the middle zone of the axis (Figure 6). The linear regression procedure proposed in [44] is applied for color-to-velocity conversion, allowing the reconstruction of the velocity profile associated with each segment (Figure 7). The peak velocity of each profile is assessed, and consequently, the mean peak velocity value *v_color,i_* (*i* = 1, …, *N*) is computed together with the standard deviation. The latter is combined with the uncertainty contribution related to the linear regression procedure [44]. These computations are repeated for all the *N* average images, obtaining an overall mean peak velocity value *v_color_* and the corresponding standard deviation retrieved through the uncertainty propagation law. All the processing steps described above are repeated for two different constant flow rate regimes (*Q*_1_ and *Q*_2_) set on the flow phantom, therefore determining *v_color,Q_*_1_ and *v_color,Q_*_2_. Finally, AMVS parameter is assessed as follows:

(5) $$\mathrm{AMVS}=\frac{\Delta v_{color}}{\Delta v_{th}}$$

where Δ*v_color_* is the difference between *v_color,Q_*_1_ and *v_color,Q_*_2_, while Δ*v_th_* is the difference between the corresponding theoretical maximum flow velocities (*v_th,Q_*_1_ and *v_th,Q_*_2_) provided by the phantom. On the other hand, AMVS standard deviation *σ*_AMVS_ is estimated through the uncertainty propagation law as follows:

(6) $$\sigma_{\mathrm{AMVS}}=\mathrm{AMVS}\sqrt{{\left(\frac{\sigma_{\Delta vcolor}}{\Delta v_{color}}\right)}^{2}+{\left(\frac{\sigma_{\Delta vth}}{\Delta v_{th}}\right)}^{2}}$$

where the two contributions in the square sum are the relative standard deviations of Δ*v_color_* and Δ*v_th_*. According to Equation (5), the dimensionless parameter is expected to be 1, i.e., maximum system sensitivity.

The method specifications assumed in this study are listed in Table 6.

#### 2.2.4. Velocity Measurements Accuracy

**Figure 8:**
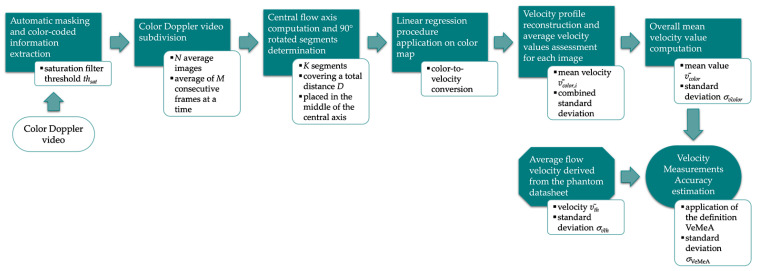
Flow chart of the image analysis-based method for VeMeA parameter estimation.

Velocity Measurements Accuracy (VeMeA) was derived from a QC test already proposed in literature [16], i.e., the mean velocity estimation, which provides an assessment of the system accuracy in the mean scatterer velocity estimation. In this study, a novel image analysis method is proposed and investigated for the automatic estimation of the test parameter through the post-processing of Color Doppler videos. The main steps of the method, implemented through an ad hoc algorithm in MATLAB, are described in the following and shown in Figure 8.

The post-processing steps of the novel method are based on the previous one for AMVS parameter assessment. In fact, the following operations are replicated: automatic masking (US image extraction), threshold-based saturation filtering *th_sat_*, averaging of *M* consecutive frames to obtain *N* average images, computation of the central flow axis as well as *K* segments rotated at 90° covering a total distance *D* and placed in the middle zone of the axis (Figure 6). Then, the linear regression procedure [44] is applied for color-to-velocity conversion of the color map, allowing the reconstruction of the velocity profile associated with each segment (Figure 7). At this point, the average velocity of each profile is assessed and, consequently, the mean velocity value ${\bar{v}}_{color,i}$ (*i* = 1, …, *N*) is computed together with the standard deviation, which, in turn, is combined with the uncertainty contribution related to the linear regression procedure [44]. These computations are repeated for all the *N* average images, obtaining an overall mean velocity value ${\bar{v}}_{color}$ and the corresponding standard deviation $\sigma_{\bar{v}color}$. Finally, VeMeA parameter is estimated as follows:

(7) $$\mathrm{VeMeA}=\frac{\left|{\bar{v}}_{color}-{\bar{v}}_{th}\right|}{{\bar{v}}_{th}}$$

where ${\bar{v}}_{th}$ is the corresponding theoretical average flow velocity provided by the Doppler phantom. As regards the standard deviation of the parameter (*σ*_VeMeA_), it is estimated by applying the uncertainty propagation law as follows:

(8) $$\sigma_{\mathrm{VeMeA}}=\mathrm{VeMeA}\sqrt{{\left(\frac{\sigma_{\bar{v}color}}{{\bar{v}}_{color}}\right)}^{2}+{\left(\frac{\sigma_{\bar{v}th}}{{\bar{v}}_{th}}\right)}^{2}}$$

where $\sigma_{\bar{v}th}$ is the flow velocity standard deviation derived from the phantom datasheet. According to the definition proposed, VeMeA is a dimensionless parameter that is expected to be as close as possible to 0, i.e., high system accuracy.

The method specifications assumed in this study are listed in Table 6.

#### 2.2.5. Temporal Resolution

**Figure 9:**
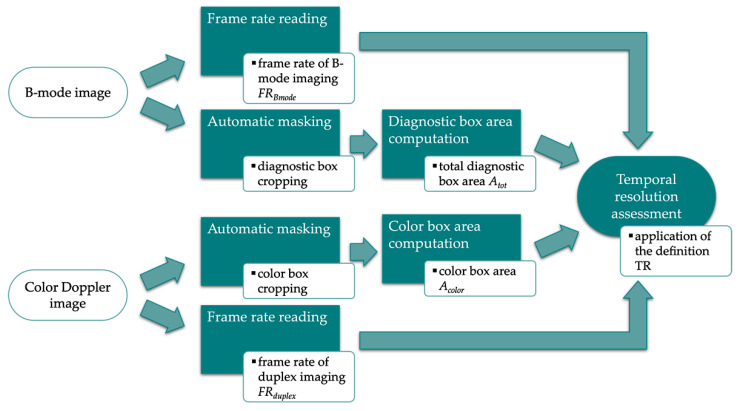
Flow chart of the image analysis-based method for temporal resolution parameter estimation.

Temporal Resolution (TR) is the minimum temporal interval for which two distinct events can be identified. Since flow changes can occur very rapidly, TR was included among the recommended QC measurements [16]. In this study, a novel image analysis method is proposed and investigated as a first attempt for the automatic estimation of CD temporal resolution related to the US system settings. The main steps of the method, developed in MATLAB environment, are described in the following and shown in Figure 9.

The method requires the processing of a Color Doppler and a B-mode image collected on a Doppler flow phantom by maintaining the probe still and the US setting constant.

Firstly, automatic masking is applied both to the grayscale and Color image to extract the diagnostic and color box, respectively, allowing the computation of the total diagnostic area *A_tot_* as well as the color box area *A_color_*. In particular, *A_color_* is retrieved by paying attention not to include the pixels belonging to the box perimeter. Then, the temporal resolution parameter is estimated by applying the following mathematical expression:

(9) $$TR=\frac{FR_{duplex}}{FR_{Bmode}}\cdot \frac{A_{color}}{A_{tot}}$$

where *FR_duplex_* is the frame rate of the duplex imaging (CD imaging superimposed on the B-mode one), while *FR_Bmode_* is the frame rate of the grayscale image only. According to the definition, when the color box is adjusted so as to include all the diagnostic box area (*A_color_*/*A_tot_* = 1), the ratio *FR_duplex_*/*FR_Bmode_* is expected to reach a maximum of 0.5. This assumption is based on the hypothesis that, under maximum system performance conditions, the frame rate of the duplex system may be half that of the B-mode imaging due to the computational cost of the Doppler processing.

### 2.3. Data Normalization

Normalization is an essential step in data analysis. Since the optimal value was different for each proposed test parameter (Table 7), a normalization procedure to extract comparable values for all QA parameters was considered.

Specifically, normalized values were expected to be in the range [0, 1], while the gold standard was 1 for all parameters. Normalized values for blind angle, registration error, average maximum velocity sensitivity, velocity measurements accuracy and temporal resolution were computed as follows:

(10) $$BA^{*}=1-\frac{BA}{BA_{\lim}}$$

(11) $$RE^{*}=1-\frac{RE}{100}$$

(12) $${\mathrm{AMVS}}^{*}=1-\left(\left|\mathrm{AMVS}-1\right|\right)$$

(13) $${\mathrm{VeMeA}}^{*}=1-\mathrm{VeMeA}$$

(14) $$TR^{*}=\sqrt{\frac{TR}{TR_{opt}}}$$

where the symbol (*) denotes the normalized value for each test parameter. In particular, *BA_lim_* in Equation (10) is the maximum expected *BA* value that in this study was assumed equal to 45°, while *TR_opt_* in Equation (14) indicates the optimal value of *TR*. The square root in the *TR* normalization was chosen in order to increase the dynamic of this parameter and appreciate small differences among *TR* results.

## 3. Monte Carlo Simulation

The measurement uncertainty contribution due to the image analysis-based methods was estimated through the Monte Carlo Simulation [47], a proper and robust tool already experienced in previous studies [43,48,49]. An MCS with 10^4^ iterations was run for each combination of test parameters, US systems, and probes, as well as configuration and phantom settings. The standard deviation (SD) from each MCS was then estimated and combined with the corresponding repeatability SD retrieved in Section 2.2.

Uniform distributions, expressed as mean ± SD, were assigned to the variables influencing the assessment of the QC parameters investigated in this study (Table 8). In the MCSs involving Color Doppler video processing, both the number of average images *N* and the number of averaged frames *M* were maintained constant throughout the iterations, while the frames to be averaged were randomized at each cycle without repetition among all the frames acquired in 3 s.

The distributions for the blind angle assessment were assigned in an analogous way to [35], while those for the registration error assessment also included an input distribution associated with the brightness filter threshold whose standard deviation *σ_b_* was set to 6% of the mean value *μ_b_*. On the other hand, the same distributions were used for the assessment of both AMVS and VeMeA parameters. Finally, for temporal resolution parameter assessment, uniform distributions were assigned to the quantities in Equation (9), assuming for both *A_color_* and *A_tot_* a standard deviation set to 3% of the corresponding mean value.

## 4. Results

Experimental outcomes for each combination of test parameters, US systems, and probes, as well as configuration and phantom settings, are reported as mean ± SD in Table 9, Table 10, Table 11, Table 12 and Table 13. Standard deviations were computed by combining *σ*_BA_, *σ*_RE%_, *σ*_AMVS_ and *σ*_VeMeA_ values with the corresponding ones estimated from MCSs. As regards the TR parameter, standard deviations were retrieved directly from the data distributions.

From blind angle outcomes (Table 9), it can be noticed that the tested phased probes showed global compatibility between the two configuration settings by considering the same flow regime. Such compatibility was no longer guaranteed for the convex array probes, for which higher BA results were retrieved in configuration A than in B. Moreover, the results obtained for both the probes of system one showed, as expected, a decreasing trend for increasing flow rates, while a reversed trend was found for the convex array probe of system three in configuration B. As per system two in configuration A, the mean value retrieved at medium flow regime *Q_M_* was higher than the one at high flow regime *Q_H_* for both the probes, and the same behavior was also found for the phased array probe of system three. Finally, for the convex probe of system three in configuration A, blind angle results were compatible and did not show a specific trend.

As regards the percentage registration error, the outcomes obtained (Table 10) for the phased array probes globally showed an increasing trend for increasing flow rates, while a well-defined behavior cannot be inferred for the convex array probes. Furthermore, RE_%_ results for system one were the closest to 0% among all three phased probe-system pairs in both configurations. On the other hand, system three, equipped with the convex array probe, showed results closer to the optimal value in configuration A only, probably due to the higher wall filter setting (Table 2) included in its clinical preset.

AMVS outcomes (Table 11) were retrieved among velocities belonging to the same flow regime, maintaining a fixed flow step of 1.5 mL·s^−1^, as listed in Table 3. They show a similar behavior between the two configurations independently of the US system for both probe models. The lowest sensitivity values that significantly deviate from one were obtained with the phased array probe of system two at a high flow rate regime *Q_H_*.

By focusing on VeMeA outcomes (Table 12), an increasing trend for increasing flow rates was found for all the convex array probes, while a distinct behavior cannot be inferred for the phased array ones. They were generally compatible between configurations A and B, and for system one was noticed that the results obtained for the convex probe were always lower than the corresponding ones for the phased probe. On the other hand, independently of the probe model, system two showed a higher occurrence of results closest to the optimal value.

Finally, temporal resolution results (Table 13) obtained for both probe models of all US systems showed, as expected, a decreasing trend for increasing Color Doppler line density setting. Moreover, by comparing each outcome in configuration A with the corresponding one in configuration B, higher TR values were always found in the latter configuration. This could probably be due to the reduction of both pre- and post-processing settings. Best outcomes (closest to 0.5) were found for system one and system two with the phased and convex array probes, respectively.

Experimental results were normalized according to the normalization steps described in Section 2.3 to allow the combination of the five test parameters retrieved for each probe at the same phantom and system settings. This allowed their representation on Kiviat diagrams and the direct comparison with the gold standard for which all the normalized QA test parameters were set to one. Therefore, the area of each polygon was computed and used as an index to quantify the overall Doppler performance of the US systems depending on the probe-configuration pair: the greater the polygon area, the higher the Doppler system performance. For ease of interpretation, the areas of the diagrams were normalized with respect to the total area of the gold standard. In this perspective, the normalized area was expected to be as close as possible to one.

**Figure 10:**
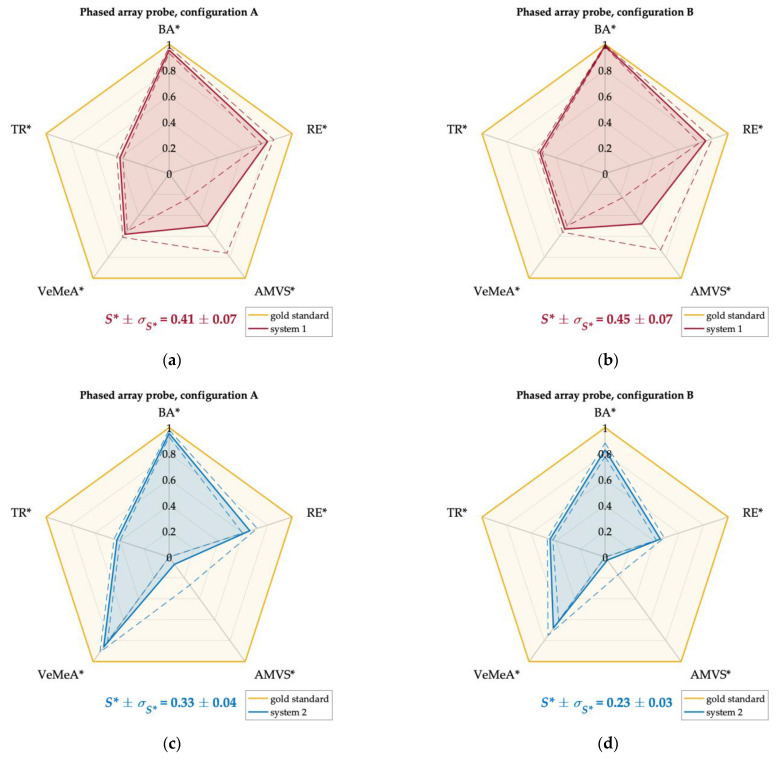

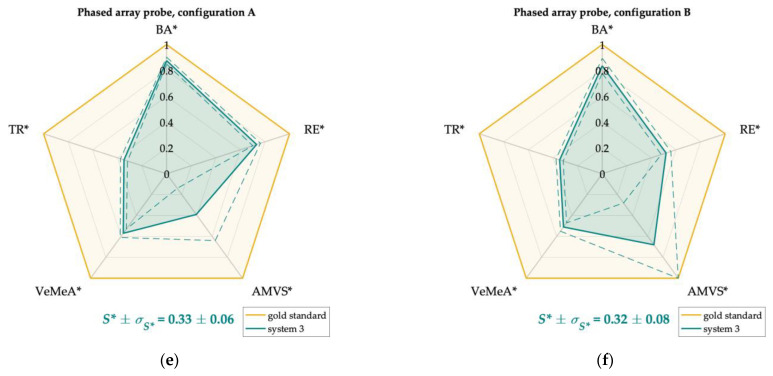
Kiviat diagrams for systems 1 (**a**,**b**), 2 (**c**,**d**) and 3 (**e**,**f**) equipped with phased array probes in configurations A (**a**,**c**,**e**) and B (**b**,**d**,**f**), for high flow rate regime *Q_H_*. Each polygon area was normalized with respect to the gold standard one.

**Figure 11:**
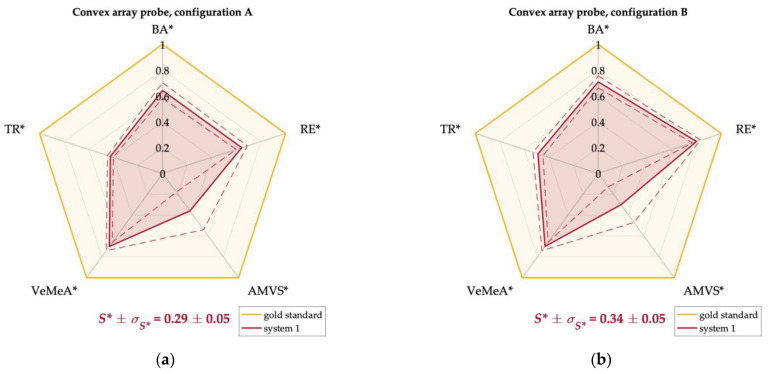

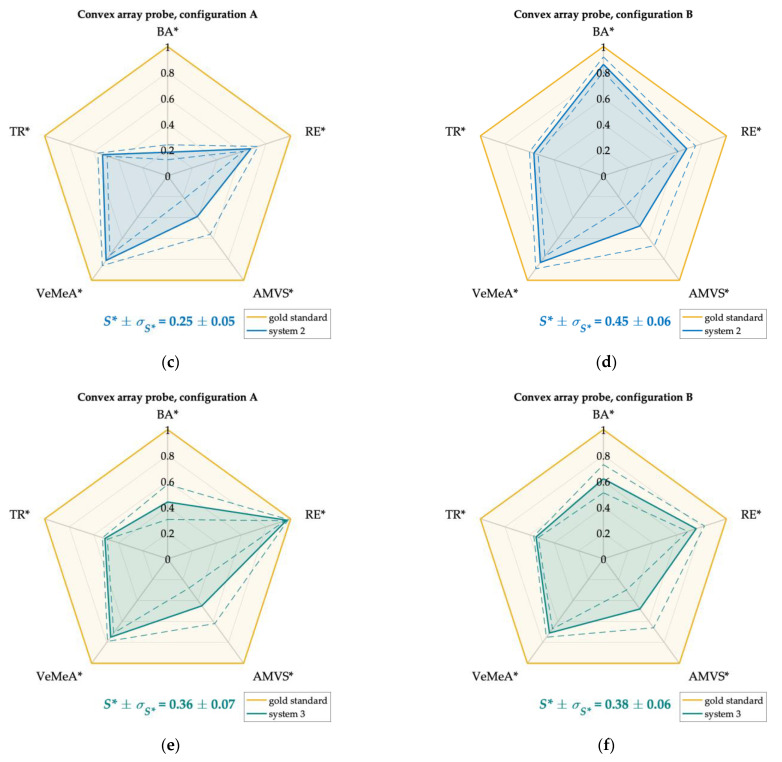
Kiviat diagrams for systems 1 (**a**,**b**), 2 (**c**,**d**) and 3 (**e**,**f**) equipped with convex array probes in configurations A (**a**,**c**,**e**) and B (**b**,**d**,**f**), for medium flow rate regime *Q_M_*. Each polygon area was normalized with respect to the gold standard one.

Kiviat diagrams for systems one, two, and three equipped with phased and convex array probes in configurations A and B are shown in Figure 10 and Figure 11. In particular, the QA parameters retrieved at a high flow rate *Q_H_* (Table 14) were used for the diagrams plot of the phased array probes since this model, preferred for echocardiography, is designed to detect high blood velocities [1]. On the other hand, the QA parameters at medium flow rate *Q_M_* (Table 15) were used for the diagrams plot of the convex array probes since this model is typically designed for abdominal imaging [1]. As regards the TR parameter, results obtained at medium CD line density setting *LD_M_* were considered for both probe models. Alongside the Kiviat diagram plot, the normalized mean area S* and the corresponding standard deviation σ_S*_ were computed (Figure 10 and Figure 11). The latter was estimated through the error propagation law.

Finally, as regards the normalized areas (Table 14 and Table 15), compatible performance was found between the two configurations for both probe models of systems one and three. As regards system two, a higher area was obtained in configurations A and B for the phased and convex array probes, respectively. By focusing on the phased array probes, system one showed the highest diagram area independently of the configuration setting (0.41 ± 0.07 and 0.45 ± 0.07 in A and B, respectively), while the lowest one was found for system two in configuration B (0.23 ± 0.03). On the other hand, the highest and lowest areas for the convex array probes were found for system two in configuration B (0.45 ± 0.06) and A (0.25 ± 0.05), respectively.

## 5. Discussion

The present study is proposed as a first approach to the combination of five Doppler test parameters based on the Kiviat diagram to quantify the performance of the US systems according to a probe-setting pair. As a first attempt, the diagram area normalized with respect to the gold standard was assumed as an index of the overall Color Doppler system performance. The assessed parameters were the blind angle, registration error, average maximum velocity sensitivity, velocity measurements accuracy, and temporal resolution. They were objectively assessed through custom-written image analysis-based methods and procedures (Figure 1, Figure 3, Figure 5, Figure 8 and Figure 9) and then normalized in the same range for the graphical representation. Three brand-new ultrasound systems, equipped with a phased and convex array probe each, were tested in two configuration settings at different flow rate regimes set on a Doppler reference device (Table 2 and Table 3).

As regards the results obtained for each single test parameter (Table 9, Table 10, Table 11, Table 12 and Table 13), it should be noticed that independently of the US system tested, BA outcomes retrieved for the phased array probes were the closest to the optimal value. By comparing the US systems, better results (closest to 0) were found for both probe models of system one independently of the configuration setting. By focusing on the percentage registration error, the phased array probes globally showed better results (closest to 0) with respect to the convex array one for both systems one and two in configuration A. Moreover, independently of the configuration, RE_%_ results for system one were the closest to the optimal value among all three phased probe-system pairs. On the other hand, AMVS results obtained for the probes of the three US systems are globally compatible among them at both configurations. However, it should also be noted that the sensitivity index is the one showing the highest SD values among the proposed QA test parameters. By considering the VeMeA parameter, the results were generally compatible between the two configurations and independent of the probe model. Lastly, temporal resolution results for both probe models of all US systems always showed, as expected, higher TR values in configuration B, probably due to the reduction of pre- and post-processing settings. Best outcomes (closest to 0.5) were found for system one and system two with the phased and convex array probes, respectively. Moreover, SD values were almost constant for all the tested phased probes in both configurations, while a limited increment was found for some convex probes.

The use of Kiviat diagrams allowed combining the quality parameters (Figure 10 and Figure 11) and estimating a single index (normalized diagram area) that provided a more immediate assessment of the CD system quality. QA parameters assessed at high and medium flow rates were used for the diagrams plot of the phased and the convex array probes, respectively. Conversely, temporal resolution results at a medium number of CD scan lines were considered for both probe models. For these cases, the outcomes (Table 14 and Table 15) confirmed that a higher polygon area was found for the probe-system pair showing higher values of the test parameters discussed above (e.g., phased array probe of system one in both configurations). Moreover, diagrams with comparable areas corresponded to US systems whose test parameters showed compatibility. These aspects suggest that the Kiviat diagram may be a useful tool for US system assessment since it seems to be directly related to the system performance. Globally, the normalized areas did not show, as expected, significant discrepancies among them since the US systems tested in this study were all brand-new systems at the same technology level. As a last remark, it should be noted that the normalized area of the diagram, together with its shape, has the advantage of preserving the relationship among the test parameters with respect to other mathematical operators, such as the arithmetic or geometric mean of the test parameters. Moreover, the Kiviat plot could provide the technician with a quick overview of the values of the single parameters highlighting both weaknesses and strengths of the Doppler system under testing and allowing the US system performance monitoring over time.

Despite the promising results, the present study is a first attempt at the use of the Kiviat diagram applied to QCs of Doppler equipment. Therefore, further investigations should be performed to assess the sensitivity as well as determine the specificity of the proposed approach. In particular, studies aimed to estimate how much the variation (due to the US system deterioration) of one (or more) of the quality parameters affects the diagram area are going to be carried out. On the other hand, US systems that have been used in the clinical setting for a few years should be tested, and the areas of their Kiviat diagrams should be compared with the ones retrieved for brand-new systems at the same technology level. This could be useful to understand whether the proposed approach is able to detect significant discrepancies among the areas of the diagrams due to an objectively evident state of deterioration. As a last remark, further investigations may include the deepening of the relationship among the QA parameters and how it could affect the shape of the Kiviat diagram.

## 6. Conclusions

Quality assessment is necessary in the US field, as for any other medical imaging equipment, in order to maintain image quality in accordance with the manufacturer’s recommendations, ensure both patient and operator safety, and comply with regulatory and accreditation requirements. Since Color Doppler is among the most used and widespread Doppler techniques in diagnostic imaging, the need for an internationally accepted quality standard for Doppler equipment is deemed important. In this regard, the study herein proposed would give a contribution to this field by investigating a first approach involving the use of the Kiviat diagram applied to QCs of Doppler equipment. Five QA parameters were objectively assessed through the post-processing of CD data, and after a normalization process, they were combined together to be represented within a single plot and summarized in a representative index. On the basis of the promising outcomes obtained and their limitations, further studies are going to be carried out for a thorough characterization of the proposed approach, including a higher number of US diagnostic systems and probe models (e.g., linear array probes).

## Figures and Tables

**Table 1:** Doppler flow phantom: technical specifications.

Parameter	Specification
Phantom model	Doppler 403^TM^ flow phantom
Scanning surface	patented composite film
Attenuation coefficient	0.70 ± 0.05 dB·cm^−1^·MHz^−1^
TMM ^(1)^	patented high equivalence (HE) gel^TM^
TMM sound speed	1540 ± 10 m·s^−1^
BMF ^(2)^ sound speed	1550 ± 10 m·s^−1^
Flow rates	customizable, constant and pulsatile
Flow measurement range	(1.7–12.5) ± 0.4 mL·s^−1^
Horizontal vessel	5.0 ± 0.2 mm inner diameter at 2 cm depth
Diagonal vessel	5.0 ± 0.2 mm inner diameter at 40° from 2 to 16 cm deep

^(1)^ TMM: Tissue Mimicking Material; ^(2)^ BMF: Blood Mimicking Fluid.

**Table 2:** Main B-mode and Color Doppler configuration settings according to the US system.

B-Mode Setting		Configuration A			Configuration B		
		System One	System Two	System Three	System One	System Two	System Three
B-mode frequency		resolution	resolution	resolution	resolution	resolution	resolution
Spatial compound imaging		ON	ON	ON	OFF	OFF	OFF
Field of view (cm)	FOV_1_	12	12	12	12	12	12
	FOV_2_	5	P: 4, C: 5	P: 4, C: 6	5	P: 4, C: 5	P: 4, C: 6
Video duration (s)		3	3	3	3	3	3
Frames resolution (px × px)		576 × 1024	920 × 1260	480 × 640	576 × 1024	920 × 1260	480 × 640
**Color Doppler setting**							
Nominal frequency (MHz)	P	2.0	2.0	2.0	2.0	2.0	2.0
	C	2.3	2.0	2.3	2.3	2.0	2.3
Wall filter	P	medium	medium	medium	minimum	minimum	minimum
	C	medium	medium	maximum	minimum	minimum	minimum
Smoothing	P	medium	medium	minimum	minimum	minimum	minimum
	C	medium	maximum	medium	minimum	minimum	minimum
Color write priority		maximum	maximum	maximum	maximum	maximum	maximum
Line density		medium	medium	medium	low	low	low

**Table 3:** Flow rate regimes settings according to the test parameter.

QA Test Parameter	Flow Mode	Flow Rate (mL·s^−1^)		
		Low *Q_L_*	Medium *Q_M_*	High *Q_H_*
Blind angle	constant	2.0	6.0	10.0
Registration error	constant	2.0	6.0	10.0
Average maximum velocity Sensitivity	constant	2.5; 4.0	7.0; 8.5	10.0; 11.5
Velocity measurements accuracy	constant	2.5	7.0	11.5
Temporal resolution	constant	–	6.0	–

**Table 4:** Variables setting for blind angle parameter estimation.

Variable	Symbol	Setting
Saturation filter threshold	*th_sat_*	0.35
Number of average images	*N*	6
Number of averaged frames ^(1)^	*M*	P: 5, C: 4
Median filter kernel	*k*-by-*k*	4-by-4 px
Number of parallel flow axis	*F*	3
Flow axis distance	*d*	1 mm
Blind threshold ^(2)^	*th_blind_*	10

^(1)^ *M* computed as *n_fr_*/*N*; ^(2)^ assumed as the smallest gray level difference distinguishable from the human eye [43].

**Table 5:** Variables setting for registration error parameter estimation.

Variable	Symbol	Setting
Brightness filter threshold	*th_b_*	*μ_b_* ^(1)^
Saturation filter threshold	*th_sat_*	0.35
Number of average images	*N*	6
Number of averaged frames ^(2)^	*M*	P: 5, C: 4
Blind threshold ^(3)^	*th_blind_*	10

^(1)^ *μ_b_* varies proportionally to the mean brightness *V* of the image; ^(2)^ *M* computed as *n_fr_*/*N*; ^(3)^ assumed as the smallest gray level difference distinguishable from the human eye [43].

**Table 6:** Variables setting for both AMVS and VeMeA parameters estimation.

Variable	Symbol	Setting
Saturation filter threshold	*th_sat_*	0.35
Number of average images	*N*	6
Number of averaged frames ^(1)^	*M*	P: 5, C: 4
Number of rotated segments	*K*	16
Covered central axis portion	*D*	20 mm

^(1)^ *M* computed as *n_fr_*/*N*.

**Table 7:** Summary of the optimal values for each proposed test parameter.

QA Test Parameter	Acronym	Optimal Value
Blind angle	BA	0°
Percentage registration error	RE_%_	0%
Average maximum velocity sensitivity	AMVS	1
Velocity measurements accuracy	VeMeA	0
Temporal resolution	TR	0.5

**Table 8:** Distribution setting in MCSs for measurement uncertainty estimation of the image analysis method for QC test parameters assessment.

Blind Angle Assessment	Symbol	Distribution	Mean ± SD
Saturation filter threshold	*th_sat_* ± *σ_sat_*	uniform	0.35 ± 0.01
Median filter kernel	*k* ± *σ_k_*	uniform	4 ± 1 px
Flow axis distance	*d* ± *σ_d_*	uniform	1.0 ± 0.3 mm
Blind threshold	*th_blind_* ± *σ_blind_*	uniform	10 ± 1
**Registration error assessment**			
Brightness filter threshold	*th_b_* ± *σ_b_*	uniform	*μ_b_* ± 0.06*μ_b_*
Saturation filter threshold	*th_sat_* ± *σ_sat_*	uniform	0.35 ± 0.01
Blind threshold	*th_blind_* ± *σ_blind_*	uniform	10 ± 1
**AMVS and VeMeA assessment**			
Saturation filter threshold	*th_sat_* ± *σ_sat_*	uniform	0.35 ± 0.01
Covered central axis portion	*D* ± *σ_D_*	uniform	20 ± 1 mm
First segment position on the axis	*x* ± *σ_x_*	uniform	*x*_0_ ± 1 mm ^(1)^
**Temporal resolution assessment**			
Duplex imaging frame rate	*FR_duplex_* ± *σ_duplex_*	uniform	*FR_duplex_* ± 1
B-mode imaging frame rate	*FR_Bmode_* ± *σ_Bmode_*	uniform	*FR_Bmode_* ± 1
Color box area	*A_color_* ± *σ_color_*	uniform	*A_color_* ± 0.03*A_color_*
Total diagnostic area	*A_tot_* ± *σ_tot_*	uniform	*A_tot_* ± 0.03*A_tot_*

^(1)^ *x*_0_ is the generic position of the first segment on the central flow axis.

**Table 9:** Blind angle parameter results (mean ± SD).

Probe Model	Flow Rate Regime	Configuration A			Configuration B		
		System One	System Two	System Three	System One	System Two	System Three
Phased array	*Q_L_*	15.7° ± 2.1°	17.9° ± 1.6°	6.9° ± 2.1°	15.4° ± 2.3°	19.2° ± 2.0°	11.1° ± 2.6°
	*Q_M_*	9.8° ± 2.5°	21.6° ± 1.6°	12.8° ± 2.4°	12.0° ± 2.5°	18.1° ± 2.1°	13.3° ± 2.5°
	*Q_H_*	2.0° ± 1.1°	2.0° ± 1.2°	5.6° ± 1.2°	0.5° ± 0.4°	7.7° ± 2.4°	7.4° ± 2.6°
Convex array	*Q_L_*	36° ± 3°	30° ± 5°	30° ± 6°	20° ± 3°	11° ± 3°	10° ± 4°
	*Q_M_*	16.1° ± 2.7°	36.8° ± 2.6°	25° ± 6°	13.1° ± 2.1°	6.2° ± 2.6°	17° ± 5°
	*Q_H_*	5.6° ± 1.2°	9.6° ± 2.8°	27° ± 5°	4.1° ± 1.3°	2.0° ± 1.3°	28° ± 5°

**Table 10:** Percentage registration error parameter results (mean ± SD).

Probe Model	Flow Rate Regime	Configuration A			Configuration B		
		System One	System Two	System Three	System One	System Two	System Three
Phased array	*Q_L_*	(3.2 ± 1.9)%	(3.3 ± 1.2)%	(12 ± 8)%	(6.2 ± 2.1)%	(12.0 ± 2.8)%	(7.9 ± 2.3)%
	*Q_M_*	(9.4 ± 2.9)%	(25 ± 14)%	(13 ± 6)%	(15 ± 3)%	(33 ± 12)%	(28 ± 7)%
	*Q_H_*	(20 ± 5)%	(34 ± 6)%	(27 ± 3)%	(18 ± 5)%	(55.1 ± 3.2)%	(48 ± 4)%
Convex array	*Q_L_*	(25.2 ± 2.2)%	(15.3 ± 0.4)%	(4.8 ± 1.4)%	(22.3 ± 1.4)%	(12.6 ± 0.9)%	(15.0 ± 2.8)%
	*Q_M_*	(36 ± 5)%	(32 ± 6)%	(3.2 ± 1.4)%	(20 ± 3)%	(32 ± 7)%	(25 ± 7)%
	*Q_H_*	(34.3 ± 1.7)%	(20 ± 3)%	(23 ± 3)%	(36.4 ± 1.8)%	(16 ± 3)%	(30 ± 7)%

**Table 11:** Average Maximum Velocity Sensitivity parameter results (mean ± SD).

Probe Model	Flow Rate Regime	Configuration A			Configuration B		
		System One	System Two	System Three	System One	System Two	System Three
Phased array	*Q_L_*	0.44 ± 0.12	0.55 ± 0.15	0.44 ± 0.12	0.41 ± 0.11	0.55 ± 0.15	0.58 ± 0.15
	*Q_M_*	0.51 ± 0.21	0.59 ± 0.42	0.29 ± 0.16	0.53 ± 0.21	0.43 ± 0.36	0.39 ± 0.18
	*Q_H_*	0.50 ± 0.26	0.07 ± 0.20	0.39 ± 0.25	0.48 ± 0.25	0.03 ± 0.14	0.68 ± 0.40
Convex array	*Q_L_*	0.54 ± 0.14	0.62 ± 0.16	0.48 ± 0.12	0.45 ± 0.12	0.60 ± 0.15	0.46 ± 0.12
	*Q_M_*	0.36 ± 0.18	0.39 ± 0.17	0.45 ± 0.17	0.30 ± 0.17	0.48 ± 0.19	0.48 ± 0.18
	*Q_H_*	0.33 ± 0.22	0.57 ± 0.27	0.55 ± 0.27	0.33 ± 0.25	0.43 ± 0.31	0.54 ± 0.45

**Table 12:** Velocity Measurements Accuracy parameter results (mean ± SD).

Probe Model	Flow Rate Regime	Configuration A			Configuration B		
		System One	System Two	System Three	System One	System Two	System Three
Phased array	*Q_L_*	0.38 ± 0.07	0.18 ± 0.12	0.38 ± 0.08	0.44 ± 0.06	0.20 ± 0.12	0.17 ± 0.09
	*Q_M_*	0.50 ± 0.04	0.12 ± 0.07	0.34 ± 0.04	0.49 ± 0.04	0.25 ± 0.07	0.33 ± 0.04
	*Q_H_*	0.42 ± 0.03	0.14 ± 0.05	0.43 ± 0.04	0.47 ± 0.03	0.32 ± 0.07	0.49 ± 0.04
Convex array	*Q_L_*	0.24 ± 0.08	0.05 ± 0.11	0.20 ± 0.08	0.17 ± 0.08	0.09 ± 0.11	0.20 ± 0.08
	*Q_M_*	0.30 ± 0.04	0.19 ± 0.05	0.25 ± 0.04	0.30 ± 0.04	0.17 ± 0.06	0.29 ± 0.04
	*Q_H_*	0.39 ± 0.03	0.42 ± 0.04	0.51 ± 0.05	0.33 ± 0.04	0.40 ± 0.04	0.64 ± 0.03

**Table 13:** Temporal resolution parameter results (mean ± SD).

Probe Model	CD Line Density	Configuration A			Configuration B		
		System One	System Two	System Three	System One	System Two	System Three
Phased array	*LD_L_*	0.10 ± 0.01	0.10 ± 0.01	0.07 ± 0.01	0.17 ± 0.01	0.12 ± 0.01	0.08 ± 0.01
	*LD_M_*	0.08 ± 0.01	0.09 ± 0.01	0.06 ± 0.01	0.14 ± 0.01	0.10 ± 0.01	0.06 ± 0.01
	*LD_H_*	0.06 ± 0.01	0.08 ± 0.01	0.05 ± 0.01	0.11 ± 0.01	0.09 ± 0.01	0.06 ± 0.01
Convex array	*LD_L_*	0.10 ± 0.01	0.18 ± 0.02	0.17 ± 0.01	0.14 ± 0.02	0.21 ± 0.02	0.19 ± 0.01
	*LD_M_*	0.09 ± 0.01	0.14 ± 0.02	0.13 ± 0.01	0.12 ± 0.02	0.16 ± 0.02	0.15 ± 0.01
	*LD_H_*	0.07 ± 0.01	0.12 ± 0.02	0.11 ± 0.01	0.10 ± 0.02	0.14 ± 0.02	0.12 ± 0.01

**Table 14:** Normalized QA test parameters and Kiviat diagram areas (mean ± SD) for systems 1, 2 and 3 equipped with phased array probes in configurations A and B at high flow regime.

US System	Configuration	BA*	RE*	AMVS*	VeMeA*	TR*	S* ± σ_S*_
1	A	0.96 ± 0.02	0.80 ± 0.05	0.50 ± 0.26	0.58 ± 0.03	0.40 ± 0.02	0.41 ± 0.07
	B	0.99 ± 0.01	0.82 ± 0.05	0.48 ± 0.25	0.53 ± 0.03	0.53 ± 0.02	0.45 ± 0.07
2	A	0.95 ± 0.03	0.66 ± 0.06	0.07 ± 0.20	0.86 ± 0.05	0.42 ± 0.02	0.33 ± 0.04
	B	0.83 ± 0.05	0.45 ± 0.03	0.03 ± 0.14	0.68 ± 0.07	0.45 ± 0.02	0.23 ± 0.03
3	A	0.86 ± 0.03	0.73 ± 0.03	0.39 ± 0.25	0.57 ± 0.04	0.35 ± 0.03	0.33 ± 0.06
	B	0.84 ± 0.06	0.52 ± 0.04	0.68 ± 0.40	0.51 ± 0.04	0.35 ± 0.03	0.32 ± 0.08

**Table 15:** Normalized QA test parameters and Kiviat diagram areas (mean ± SD) for systems 1, 2 and 3 equipped with convex array probes in configurations A and B at medium flow regime.

US System	Configuration	BA*	RE*	AMVS*	VeMeA*	TR*	S* ± σ_S*_
1	A	0.64 ± 0.06	0.64 ± 0.05	0.36 ± 0.18	0.70 ± 0.04	0.42 ± 0.02	0.29 ± 0.05
	B	0.71 ± 0.05	0.80 ± 0.03	0.30 ± 0.17	0.70 ± 0.04	0.49 ± 0.04	0.34 ± 0.05
2	A	0.18 ± 0.06	0.68 ± 0.06	0.39 ± 0.17	0.81 ± 0.05	0.53 ± 0.04	0.25 ± 0.05
	B	0.86 ± 0.06	0.68 ± 0.07	0.48 ± 0.19	0.83 ± 0.06	0.56 ± 0.04	0.45 ± 0.06
3	A	0.44 ± 0.14	0.97 ± 0.01	0.45 ± 0.17	0.75 ± 0.04	0.51 ± 0.02	0.36 ± 0.07
	B	0.62 ± 0.11	0.75 ± 0.07	0.48 ± 0.18	0.71 ± 0.04	0.55 ± 0.02	0.38 ± 0.06

## Data Availability

Not applicable.
